# Telehealth Models for PrEP Delivery: A Systematic Review of Acceptability, Implementation, and Impact on the PrEP Care Continuum in the United States

**DOI:** 10.1007/s10461-024-04366-3

**Published:** 2024-06-10

**Authors:** Stephen Bonett, Qian Li, Anna Sweeney, Denise Gaither-Hardy, Hussein Safa

**Affiliations:** 1 School of Nursing, University of Pennsylvania, 418 Curie Blvd, Philadelphia, PA 19104 USA; 2 Lincoln University, Lower Oxford Township, PA USA; 3 Albert Einstein Healthcare Network, Philadelphia, PA USA

**Keywords:** Pre-exposure Prophylaxis, Telehealth, HIV Prevention, Health Equity, Access to care

## Abstract

Pre-exposure prophylaxis (PrEP) is pivotal in curbing HIV transmission and is integral to the national plan to end the HIV epidemic in the United States (US). Nonetheless, widespread PrEP adoption faces barriers. Telehealth delivery models for PrEP, or telePrEP, can enhance PrEP access and adherence by providing flexible care remotely. This study presents a systematic review of telePrEP programs in the US, aiming to describe model characteristics and summarize clinical, implementation, and equity outcomes. We reviewed studies published from 2012 to 2023. We included articles that described telePrEP systems in the US and measured PrEP care continuum outcomes (awareness, initiation, uptake, adherence) or acceptability of the intervention by program users. Eight articles describing six distinct telePrEP initiatives met our inclusion criteria. Studies described models implemented in community-based, academic, and commercial settings, with most programs using a direct-to-client telePrEP model. Across studies, clients reported high acceptability of the telePrEP programs, finding them easy to use, convenient, and helpful as a tool for accessing HIV prevention services. No programs were offering injectable PrEP at the time these studies were conducted. Data was limited in measuring PrEP retention rates and the reach of services to underserved populations, including Black and Latinx communities, transgender individuals, and cis-gender women. Findings underscore the potential of telePrEP to bolster the reach of PrEP care and address structural barriers to access. As telehealth models for PrEP care gain prominence, future research should concentrate on refining implementation strategies, enhancing equity outcomes, and expanding services to include injectable PrEP.

**Supplementary Information:**

The online version contains supplementary material available at 10.1007/s10461-024-04366-3.

## Resumen

La profilaxis preexposición (PrEP) es fundamental para frenar la transmisión del VIH y es parte integral del plan nacional para acabar con la epidemia del VIH en los Estados Unidos (EE. UU.). Sin embargo, la adopción generalizada de la PrEP enfrenta barreras. Los modelos de entrega de PrEP a través de la telesalud (telePrEP) pueden mejorar el acceso y la adherencia a la PrEP al proporcionar atención medica flexible de forma remota. Este estudio presenta una revisión sistemática de la literatura sobre los programas de telePrEP en los EE. UU., con el objetivo de describir las características del modelo y resumir los resultados clínicos, resultados de implementación y resultados de equidad. Revisamos estudios publicados entre 2012 y 2023. Incluimos artículos que describían sistemas de telePrEP en los EE. UU. y medían los resultados del continuo de atención de la PrEP (conciencia, iniciación, adopción, adherencia) o la aceptabilidad de la intervención por parte de los usuarios del programa. Los ocho artículos que describen las seis iniciativas distintas de telePrEP cumplieron nuestros criterios de inclusión. Los estudios describieron modelos implementados en entornos comunitarios, académicos y comerciales, y la mayoría de los programas utilizaron un modelo de telePrEP dirigido directamente al cliente. En todos los estudios, los clientes reportaron una alta aceptabilidad hacia los programas de telePrEP, encontrándolos fáciles de usar, convenientes y útiles como herramienta para acceder a los servicios de prevención del VIH. Ningún programa ofrecía PrEP inyectable en el momento en que se realizaron estos estudios. Los datos fueron limitados para medir las tasas de retención de la PrEP y el alcance de los servicios a las poblaciones desatendidas, incluyendo las comunidades Negras y Latines, las personas transgénero y las mujeres cisgénero. Los hallazgos subrayan el potencial de la telePrEP para reforzar el alcance de la atención de la PrEP y abordar las barreras estructurales de acceso. A medida que los modelos de telesalud para la atención de la PrEP ganan protagonismo, las investigaciones futuras deberan concentrarse en refinar las estrategias de implementación, mejorar los resultados de equidad y expandir los servicios para incluir la PrEP inye`ctable.

## Introduction

Pre-exposure prophylaxis (PrEP) is a powerful tool for lowering individual risk for HIV and a pillar in the national plan to end the HIV epidemic (EHE) in the United States (US) [1]. However, significant barriers exist to achieving widespread uptake of PrEP among those at risk of acquiring HIV [2]. Specifically, limited appointment availability with PrEP providers [3], experiences of stigma and discrimination while seeking healthcare [4], and logistical barriers related to transportation and scheduling [5] have been identified as key barriers to widespread PrEP access. These multilevel barriers to accessing PrEP have resulted in disparities in PrEP uptake by race, gender, and age; Black and Latinx communities, transgender individuals, cisgender women, and young people have substantial unmet need for PrEP [6, 7]. Strategies are needed to expand the availability of PrEP for populations that face ongoing barriers to access.

Limited geographical availability of PrEP providers has resulted in significant barriers to access for communities that could benefit from PrEP. Nearly 1 in 5 PrEP-eligible men who have sex with men in the US live at least 30 min away from the nearest PrEP provider, with even more gaps in access being seen in rural and Southern areas [8]. In particular, limited access to transportation options to get to brick and mortar clinics for sexual health care and challenges related to scheduling appointments around work and other obligations have been noted as consistent barriers to engagement with PrEP services [5, 9]. Implicit biases and manifestations of discrimination against marginalized groups, including racial/ethnic minorities and sexual and gender minorities, in healthcare settings has also fostered distrust and created additional barriers to PrEP uptake [2]. Additionally, internalization of the stigma surrounding PrEP and sexual health can deter individuals who could benefit from PrEP from seeking it [10].

One of the strategies being tested to expand the availability of PrEP and address these barriers to access is through telehealth delivery of PrEP services. Telehealth models for PrEP delivery aim to improve availability and access to PrEP by providing services in a flexible and convenient format that does not require travel to a physical clinic for PrEP care. This PrEP delivery model may lower barriers to access for individuals who face transportation, scheduling, or availability constraints and address barriers related to stigma and confidentiality that keep people from seeking PrEP services in person [11]. Globally, telehealth models for PrEP services and related sexual health care have been found to be feasible and acceptable by clients and healthcare providers [12–14]. Before the COVID-19 pandemic, telehealth systems for delivering PrEP care in the US were emerging but were described in the scientific literature only in a limited number of early protocol papers and conference presentations [15, 16]. The number of telePrEP programs has increased following the pandemic, as has the systematic evaluation and reporting of these models [17].

Despite this recent increase in reporting on telehealth models for PrEP delivery, there is sparse data on the impact of these programs on clinical outcomes in the PrEP care continuum, implementation outcomes including acceptability and appropriateness, or equity outcomes (e.g., reach to marginalized communities). While some reviews on this topic exist [18–20], they are limited in scope - either excluding post-pandemic developments or lacking systematic reporting on clinical and implementation outcomes. There is a need for a comprehensive review and synthesis of the existing literature on telePrEP programs in the US and their outcomes. The present review aims to (1) to provide a detailed description of the characteristics of telePrEP models currently reported in the scientific literature in the US, (2) summarize data from these programs measuring clinical, implementation, and equity outcomes, and (3) discuss implications and directions for future research.

## Methods

### Literature Search Strategy and Data Sources

We conducted a systematic review of peer-reviewed literature on the delivery of PrEP using telehealth. We used the Preferred Reporting Items for Systematic Review and Meta-Analysis Protocols (PRISMA-P) as a guide for our review methodology [21]. We searched five major databases (i.e., PubMed, APA Psych Info, Sociological Abstracts, CINAHL, and Scopus) to identify studies that were published between January 1st 2012, the year that PrEP was approved by the Food and Drug Administration, and March 10th 2023 when our search was conducted. Our search query was created by combining search terms related to three major constructs (PrEP, telehealth, and key outcomes) using AND operators between the three constructs and OR operators within each construct (Supplemental Fig. 1: Search Strategy). The CADIMA platform, a free web-based tool for conducting and reporting the systematic review process, was used to manage references, remove duplicates, and conduct screening and study selection [22].

### Selection Criteria

Studies were eligible for inclusion in our review if they (1) described a system for delivering PrEP services through telehealth that included interaction with a prescribing provider either synchronously (e.g. video conference) or asynchronously (e.g. text-based communication with a provider), (2) reported outcomes on some aspect of the PrEP care continuum (awareness, willingness, uptake, maintenance, or adherence) or on the perceived acceptability, feasibility, or appropriateness of the system by the clients accessing the services. (3) took place in the US, (4) were reported in English, and (5) was published in a peer-reviewed journal and consists of the following study design: randomized-controlled trial, implementation trial, quasi experimental design, pre-post evaluation, mixed methods evaluation, program case study, program case series, or survey. We focused on U.S.-based programs because the regulatory, insurance, and healthcare delivery landscape in the U.S. is distinct from other global regions in ways that substantially impact PrEP implementation, and a central aim of our review was to discuss implications of findings for the U.S. Ending the HIV Epidemic initiative and related national public health goals around expanding PrEP access. Studies that only reported service utilization volume (e.g. number of visits, telehealth adoption rates) without indicators of PrEP uptake or adherence were not included in this review. Abstracts from conference proceedings, book chapters, editorials, and non-peer-reviewed literature were excluded. We excluded articles that didn’t sufficiently describe the telehealth model for PrEP delivery and articles that described general telehealth programs set up in response to the COVID-19 pandemic that weren’t specifically designed for PrEP delivery.

### Study Selection and Data Extraction

After removal of the duplicates, three authors (SB, AS, QL) screened the titles and abstracts using the established selection criteria, with at least two authors independently screening each study. Where discrepancies occurred between the two authors, all three screening authors reached consensus about whether to include the article. For articles that passed title and abstract screening, all three authors (SB, AS, QL) conducted a full-text review to determine inclusion in the final review. Any discrepancies between the determination between the three authors was resolved by discussion. Several articles that were not identified in the primary search strategy but were potentially eligible for inclusion were found by manual review of the reference lists of the included articles. These articles were passed through the same system of title and abstract review followed by full text review to determine final eligibility for inclusion.

The data extraction process was conducted by all five authors (HS, QL, AS, SB, DG). Two authors independently extracted relevant information using a standardized data extraction form. For each included article, we extracted details from studies on telehealth models for PrEP service delivery including study timeframe and location, implementing organization name and type, model for service delivery, study population and sample size, laboratory testing protocols, modalities for patient interactions with navigators and prescribing providers, PrEP modalities offered, outcomes measured, and a summary of key study findings. The model of service delivery was classified as direct-to-client, presenting site, or partner site, as described by a recent guidance document published by a CDC-funded non-profit [23]. In the direct-to-client model, the client connects with the provider from a location of their choosing, such as their home, using a computer or smartphone. The presenting site model involves two clinical sites working together, where the client is located at one site with a healthcare provider and connects via telehealth to a specialist at a remote site. Finally, the partner site model involves an organization partnering with the telehealth provider to offer a physical location where clients can go to access the telehealth equipment and conduct a virtual visit with a remote provider. AS synthesized the extracted data and resolved minor discrepancies to create a final data table for analysis. Using the final data table, QL and SB analyzed commonalities and differences shared between the 8 articles. This involved finding any potential themes within each of the predetermined details listed above and highlighting noteworthy differences based on the study population and design.

After data extraction, a quality appraisal was conducted to assess the methodological rigor of the included studies. Using the Mixed Methods Appraisal Tool [24], two authors (AS and SB) independently conducted a critical appraisal of all included studies. They met to discuss discrepancies and reached consensus on the final quality appraisal.

## Results

**Fig. 1:**
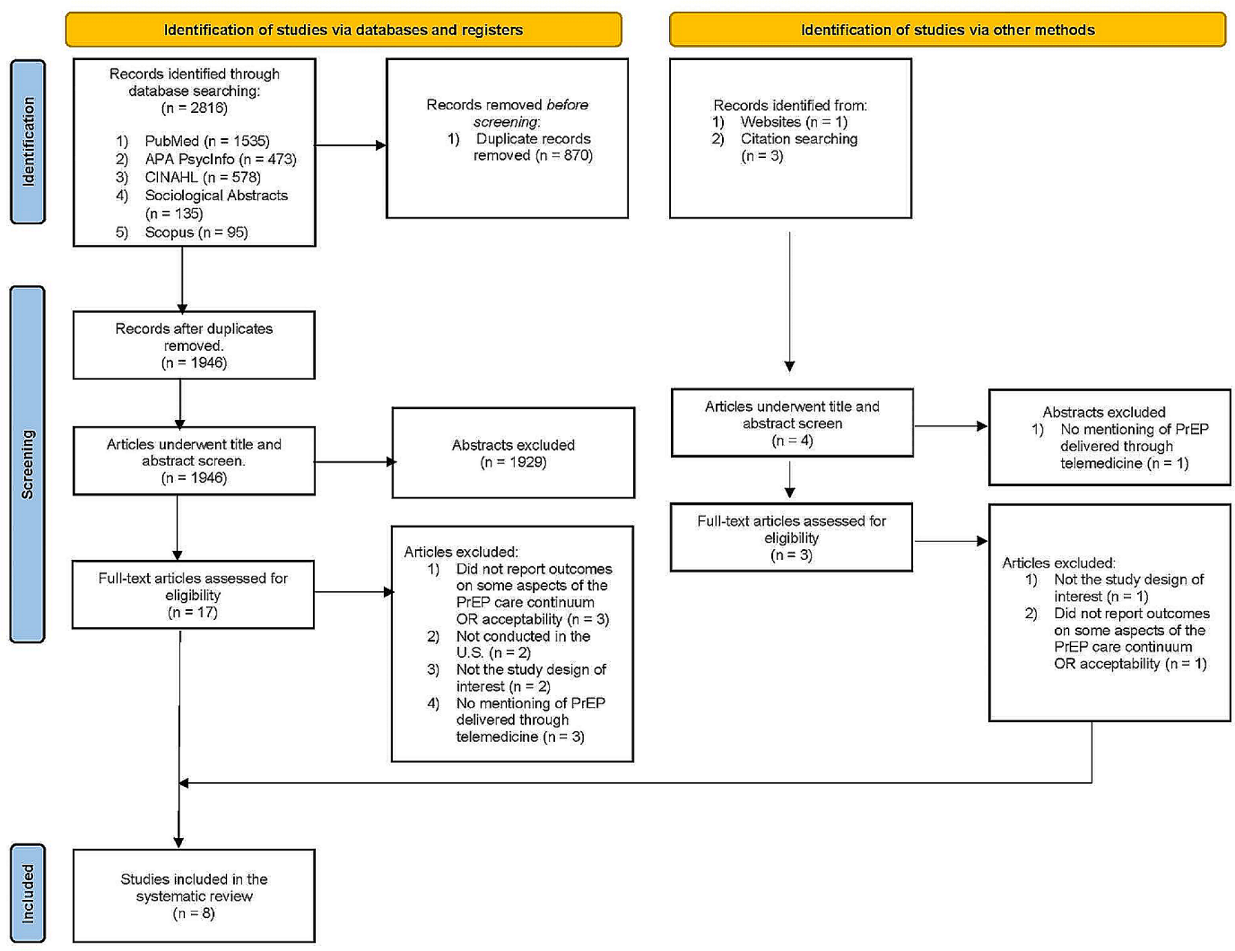
Preferred Reporting Items for Systematic Review and Meta-Analysis (PRISMA) diagram for the articles reviewed [55]

The search of the five databases yielded a total of 2,816 articles. After removing duplicate articles, 1,946 unique articles remained to be reviewed. During the title and abstract screening phase, 1,929 articles were excluded for not meeting the eligibility criteria. In the full text screening phase, 10 more articles were excluded for the following reasons: three did not report outcomes related to the PrEP care continuum or acceptability of telehealth for PrEP, three did not describe a telehealth system for delivering PrEP services, two were protocol or review papers rather than original research, and two were not conducted in the United States. Additionally, one relevant article was identified through manually searching the reference lists of the included articles. Overall, after screening, eight articles satisfied the eligibility criteria and were included in this review [25–32]. (Fig. 1)

### Quality Appraisal

Overall, seven of eight included studies met all criteria or all but one criterion for quality. Four studies used a quantitative descriptive design. Of these, one study included a sample that was not representative of the target population, and another study did not include appropriate measures to address their research question. Two qualitative studies were included, with one study reporting results that were poorly substantiated by the data and another study lacking coherence between the data and the interpretation of findings as well as failing to adequately report how findings and interpretation were derived from the data. One quantitative non-randomized study was included that failed to control for confounding factors, and one mixed methods study was included that met all criteria for quality but did not adequately describe the integration of qualitative and quantitative data. A full description of the quality appraisal can be found in Supplemental Table 1.

### Models for Telehealth Delivery of PrEP Services

A summary of the characteristics of the six distinct telePrEP programs described in the included studies can be found in Table 1. Two studies described a single program that was implemented by a commercial organization called NURX [27, 28]. One program (PrEPTECH) was implemented by an academic institution [30], one as a partnership between an academic institution and a community-based clinic [29], and one as a partnership between an academic institution and a public health department (Iowa TelePrEP), which was described by two studies [25, 26]. One program was implemented in the context of a research study called Project Empowering [31], and one was implemented in a community-based clinic called the Gay City Wellness Center [32].

**Table 1:** TelePrEP program characteristics

Program Name	Implementing Organization	Care Delivery Model	Laboratory Testing	Staff Interactions	Insurance Coverage
Iowa TelePrEP Program [25, 26]	Partnership between an academic institution and a public health department	Direct to client	In-person	Tele-Visits	Staff assisted with medication assistance program enrollment
Nurx [27, 28]	Commercial Organization	Direct to client	In-person at a laboratory local to the patient	Synchronous or asynchronous chats with staff. Synchronous interactions not required	No assistance program available; insurance not accepted for cost of visit but private insurance plans are accepted for medications [56]
Palmetto Community Center Pilot Program [29]	Partnership between an academic institution and a community-based clinic	Direct to client	In-person at community center	Three video visits; Four asynchronous e-visits	Not reported
PrEPTECH [30]	Academic Institution	Direct to client	Self-sampling for STI tests collected at home and brought to a local Quest lab. Bloodwork drawn at Quest.	Telephone visit	Medication and all study related costs covered by sponsor, Gilead
Project Empowering [31]	Research study	Partner Site	Screening for pregnancy, HIV, and Hep C done onsite. Other testing done at nearby lab	Phone or video visits with provider. In-person case managers	Medication and all study related costs covered by sponsor, Gilead
Gay City Wellness Center [32]	Community-based Clinic	Presenting Site	Collected on-site at PrEP Clinic	In person or video visit	Staff assisted with medication assistance program enrollment

Four programs used a direct-to-client telePrEP model [25–30]. One program used a presenting site model where clients at a community-based clinic were offered telehealth appointments with a physician via videoconference [32]. One program used a partner site model where participants visited a multidisciplinary research and community space for study activities and received PrEP prescriptions remotely from infectious disease physicians [31].

### Laboratory Testing Protocol

All programs except one required participants to complete in-person lab testing at a partner site, lab of choice, or the clinical site providing telePrEP services [25–29, 31, 32]. One program allowed self-collection of samples for sexually transmitted infection (STI) testing but still required in-person bloodwork [30]. None of the programs utilized mail-in lab services.

### Interaction with TelePrEP System

One program (Iowa TelePrEP) employed tele-navigators to assist participants remotely by answering questions about PrEP and assisting with connection to insurance and benefits [25, 26]. Another two programs utilized in-person case managers to help navigate the telePrEP process [31, 32]. The Nurx telehealth PrEP delivery model determined eligibility primarily through questionnaires rather than using navigators, with clients able to contact customer support if they had any questions about the process [27, 28]. The Iowa TelePrEP system allowed synchronous communication with pharmacist providers. The NURX system used asynchronous text messaging for participant questions and consultation with the provider. The remaining programs offered phone or video visits as options for interacting with the provider.

### Insurance Coverage and PrEP Modalities

Some programs had systems to assist uninsured participants with insurance enrollment or copay reimbursement. Two programs provided PrEP medication at no cost to all participants [30, 31]. All programs offered daily oral PrEP; none explicitly reported providing education on on-demand PrEP or offering long-acting injectable PrEP.

### Study Settings and Sample Characteristics

A description of the included studies’ sample characteristics, measured outcomes, and key results can be found in Table 2.The eight included studies were conducted in various geographic locations across the United States, including rural and urban regions of Iowa [25, 26]; California, New York, Florida, and Illinois [27, 28]; Charleston, South Carolina [29]; a city in New England [31]; the San Francisco Bay Area [30]; and Seattle, Washington [32].

**Table 2:** Study characteristics and summary of key findings

Study and Program	Location	Sample Size	Race/ Ethnicity	Gender	Outcomes Measured	Summary of Findings
Chasco et al. (2021) [25] Iowa Tele-PrEP	Southeast Iowa	Quantitative Sample = 258 Qualitative Sample = 27	White (83%); Black (10%); Asian (3%); Multiracial (2%); Native Amer/ Alaskan Native (1%); Other (1%). Hispanic (11%); Non-Hispanic (89%)	Men (95%); Women (3.5%); Genderfluid (1%); Transwomen (0.5%)	(1) Staff: acceptability (2) Innovation: adaptability, facilitators and barriers, success engaging target populations	Staff found this model highly acceptable and appropriate. Program reduced burden (such as travel) and increased convenience, privacy, and accessibility. Of 206 completed initial visits, 167 initiated PrEP (81%) Program improved geographical access, this improvement was not enough to adequately overcome racial disparities. Difficulties reaching Black and Latinx and rural area MSM communities persisted. Program success supports scale up and reproducibility with partnerships of other local public health departments.
Hoth et al. (2019) [26] Iowa Tele-PrEP	Southeast Iowa	127	White (81%), Black (7%), Latinx (6%), Asian (3%), Multiracial (2%), Native American (1%)	Cisgender men (91%), Cisgender women (6%), non-binary (3%)	(1) Referrals to telePrEP (2) Completion of initial video visits (3) Initiation of FTC/TDF among all clients with initial visits (4) Retention in telePrEP at 90 and 180 days following FTC/TDF initiation.	186 referrals, 127 initial visits completed within 40 days (68% of referrals) 91% (116/127) started PrEP 78% (99/127) retained at 90 days, 56% (71/127) retained at 180 days. Completion rates for guideline-based blood testing was 96%.
Hughes et al. (2021) [27] Nurx	California, New York, Illinois, and Florida	31	White (32%) African American (13%) Latinx (26%) Asian (10%) Mixed or more multiracial (19%)	Men (84%) Women (16%)	Client perceptions of acceptability and convenience of Nurx to access PrEP	Barriers: -Concerns about the privacy, confidentiality, and quality of internet-based care -Difficulty navigating program steps: -Less personal relationship with the provider Facilitators: -Decreased wait times for appointment -Online questionnaires provided a comfortable space for clients to ask sexual health questions
Koester et al. (2020) [28] Nurx	California, New York, Florida, and Illinois	26	White (32%) Latinx ( 29%) Black (16%) Multiracial (13%) Asian/Pacific Islander (10%)	Cisgender men (84%) Cisgender women (16%)	Acceptability and feasibility of in-person laboratory monitoring for telePrEP program (qualitative)	Participants reported that commercial laboratories were easy to access and convenient. They also discussed how having quarterly monitoring requirements helped them to prioritize getting regular STI testing.
Player et al. (2022) [29] Palmetto Community Center Pilot Program	Charleston, South Carolina	20	White (95%), Black (5%)	Men (100%).	(1) Attendance at follow up visit (2) Clinical: -Diagnosis of STIs -PrEP adherence (3) Client Acceptability	20 enrolled, 16 completed 6 month visit 15% had positive STI. Self-reported PrEP adherence 68-70% at first three visits, and 60% at final visit. 87.5% reported satisfaction 9–10/10, and only 6.3% rated below 6
Refugio, et al. (2019) [30] PrEPTECH	San Francisco, California	25	Latinx (40%) Asian/Pacific Islander (32%) White (16%) Black (8%) Middle Eastern (4%)	Men (100%)	(1) Client perception of acceptability and usability (2) Engagement of Target Population (3) Time to initiation	> 85% of clients agreed PREPTECH is a better way to access PrEP 88% agreed PrEPTECH site was easy to use 100% agreed PrEPTECH was fast > 85% found PrEPTECH very or extremely trustworthy Goal of > 60% racial/ ethnic minority enrollment met Average time to PrEP initiation was 46 days.
Meyer et al. (2022) [31] Project Empowering	Large City in New England	Total: 105 Index: 38 Risk Network: 67	White( 56.2%), Black (26.7%), Latinx (14.3%), Other (28.6%)	Cisgender women (70.5%), Cisgender men (30%), Transgender (1%)	(1) Feasibility and acceptability of innovation (2) Engagement in PrEP care continuum	Clients identified reduced risk, increased health, and HIV knowledge as benefits of program. Did not like frequency of study appointments or the burden of daily medication 52 individuals were PrEP eligible (49.% of 105 enrolled), 30 (58%, 30/52) were interested, 24 (46%, 24/52) agreed to start PrEP; 21 (40%) filled script, 11 (21%) refilled at mo.6.
Stekler et al. (2018) [32] Gay City Wellness Center	Seattle, Washington	10	White (20%), Asian (20%), Hispanic (30%), Other (30%)	Cisgender men (100%)	(1) Client acceptability (2) PrEP initiations and retention using tele-health vs. traditional model	Showed preliminary feasibility and acceptability. 70% (7/10) of participants were prescribed PrEP 29% (2/7) of participants completed their 3-month follow up visit

All studies focused on adult populations, with most participants being between 20 and 40 years of age. In four of the eight studies, the majority of the sample was non-Hispanic white [25, 26, 29, 31]. The remaining four studies had racially diverse samples with larger proportions of Hispanic participants and non-Hispanic white participants [27, 28, 30, 32]. Across the studies, the majority of the participants identified as cisgender men, with the exception of one study that recruited primarily cisgender women involved in the criminal justice system [31]. Three of the studies reported transgender individuals and non-binary individuals to make up about 2% of the study samples [25, 26, 31]; the remaining studies did not include gender diverse individuals. In the four studies that collected insurance status, the majority of participants were privately insured through an employer, a family member’s plan, or unspecified private insurance [26, 27, 30, 32]. Sample sizes ranged from 125 to 250 for quantitative studies and 10–40 for qualitative studies.

### Implementation Outcomes

#### Client-Level Acceptability and Feasibility

Client-level acceptability and feasibility of telePrEP programs was generally high. In one study, 87.5% of clients reported satisfaction ratings of 9 or 10 out of 10 with the telePrEP program [29]. Over 85% of clients in another study agreed that the telehealth system for PrEP delivery was a better way to access PrEP, was easy to use, and was trustworthy [30]. Clients in a study of the NURX platform reported that the program reduced the burden of medical appointments with reduced waiting times and provided a comfortable online space for clients to ask questions about sexual health [27]. However, clients in this study also expressed concerns about the privacy, confidentiality, and quality of internet-based care, including difficulty in navigating program steps like using the website, coordinating lab visits and medication shipments, and having to accept not having a personal relationship with the provider.

#### Staff-Level Acceptability and Feasibility

Staff-level acceptability and feasibility of telePrEP was high in the Iowa TelePrEP program. Staff found it highly acceptable and appropriate due to the reduced travel burden and increased convenience, privacy, and accessibility for clients [25]. They found integration into existing public health workflows successful, though some standardization is needed for scaling up the program and establishing connections with other organizations. However, staff noted persisting challenges in reaching rural, Black, and Latinx communities with telePrEP services.

### Clinical Outcomes

#### PrEP Initiation

Four studies reported data on PrEP initiation rates. In the latest report from the Iowa TelePrEP program, 81% (167/206) of individuals who completed an initial visit successfully initiated PrEP [25]. In a small pilot telePrEP program, 50% (20/40) of referred participants enrolled and were prescribed PrEP [29]. In a study focused on providing telehealth delivery of PrEP services to women involved in the justice system, 46% (24/52) of eligible individuals were prescribed PrEP [31] A small study that compared using telehealth for PrEP services to their existing in person PrEP services found that 70% (7/10) of participants using the telehealth option were prescribed PrEP [32].

#### Retention in PrEP Care

Five studies reported retention in PrEP care at least 3 months after initiation. In the Iowa TelePrEP program, 56% (71/127) of participants were retained in care at 180 days [26]. In the study reported by Player et al., 80% (16/20) of participants were retained in care at the end of the six-month study [29]. In a small pilot study of telehealth delivery of PrEP in the San Francisco area, 84% (21/25) completed a follow up visit at six months [30]. In the study focused on justice-involved women, 46% (11/24) of participants prescribed PrEP completed a medication refill at the six-month time point [31]. In the study reported by Stekler et al., only 29% (2/7) of participants who were prescribed PrEP completed their 3-month follow up visit [32].

### Equity Outcomes

In terms of reach and equity, few studies reported specific outcomes related to equity or reach of programs to marginalized communities. One study reported meeting its target of enrolling over 60% racial/ethnic minority individuals into their program, although this was a small pilot study [30]. One study reporting on the Iowa TelePrEP program discussed how their program had higher participation from Black and Latinx individuals relative to the general population in Iowa; however, reach to Black and Latinx communities still lagged when compared to their proportion among people living with HIV in Iowa. The authors suggest that this pattern indicates that the program efforts to increase geographic access, while promising, still fell short in adequately addressing racial disparities in PrEP access [25].

## Discussion

Our review identified eight studies describing six distinct examples of telehealth programs for PrEP service delivery. Most of the included studies described a direct-to-client model for service delivery, none used mail-in services for laboratory specimen, and none reported offering long-acting injectable PrEP. Overall, both clients and staff regarded these telehealth models as highly acceptable and feasible. Few studies measured or reported outcomes evaluating whether telehealth models for PrEP service delivery improved equity outcomes or expanded reach of services to historically marginalized groups.

Our finding that clients and service providers found telehealth models for PrEP delivery to be highly acceptable and feasible aligns with prior research exploring perceptions of telehealth PrEP models both globally and within the United States. For example, studies conducted in Malaysia, Brazil, and Scotland found that patients rated telehealth services for PrEP initiation and monitoring as highly acceptable [12–14]. . Additionally, a mixed methods study of PrEP-eligible adults in Mississippi found that participants endorsed the use of remote PrEP care models, citing improved accessibility and privacy [11]. Together with our review, these studies demonstrate that telePrEP is viewed as an acceptable modality by end-users across various global regions and models of care. The COVID-19 pandemic spurred rapid adoption of telehealth across health care, increasing general familiarity and comfort with virtual care options [33]. However, one study in this review found that the lack of personal interaction with a provider was described as a weakness by some patients using a commercial telePrEP program [27]. This commercial model relied entirely on asynchronous communication and did not incorporate the option for live video or phone conversations with the prescribing provider. In contrast, weaknesses related to impersonal care were not reported in studies of telePrEP programs that enabled synchronous telehealth visits or connections with navigators. Thus, incorporating live, interpersonal components into telePrEP systems may improve perceptions of quality of care and acceptability among clients and improve comfort around discussing sexual history and reduce the stigma around PrEP and sexual health. Building these personal relationships could also strengthen patient-provider rapport to support retention and continuity of care. Hybrid models blending digital convenience with options for real-time communication may balance efficiency and comprehensive support.

In studies that measured clinical outcomes, there were moderate levels of PrEP initiation and retention in these telehealth models for PrEP service delivery. PrEP initiation rates ranged from 46 to 81% across the telehealth programs reporting these metrics. These percentages are comparable to initiation rates seen in some brick-and-mortar PrEP clinics serving similar populations [34, 35]. Among the reviewed telehealth initiatives with retention data, retention at 3 or 6 months post-initiation varied substantially from 29 to 84%. While this represents significant variability in PrEP retention across the models studied, it is also comparable to reported rates of PrEP retention from in-person clinical settings in the United States [35–37]. Overall, the available data shows similar outcomes from telehealth models regarding PrEP access and persistence when compared to traditional in-person care models.

None of the telehealth PrEP delivery models in this review used mail-in lab collection of self-sampled specimens. There is a growing body of research and infrastructure being developed around systems for at home self-sampling for the labs needed for PrEP [38]. For example, researchers at Emory University have developed a system for research participants to request home self-collection kits for HIV and STI testing that can be mailed anywhere in the United States that mailed over 1,100 tests in its first two years in operation [39]. Specific home-testing protocols have also been developed as part of routine PrEP care where existing PrEP patients can complete their regular HIV and STI testing at home and mail the samples to a laboratory for processing [40]. As telePrEP systems mature, integration of mail-in self-sampling for required lab work could further increase the convenience and accessibility of these models. Allowing clients to collect their own specimens for testing at home aligns with the goals of telehealth to provide flexible, patient-centered care and could boost retention in PrEP services.

We also found that none of the reviewed telehealth systems for PrEP delivery described pathways for clients to access long-acting injectable PrEP. Long-acting injectable PrEP was approved by the US Food and Drug Administration in 2021 [41], and efforts to integrate this PrEP modality into clinical practice are currently underway [42]. As injectable PrEP becomes increasingly available, incorporating education, counseling, and access support around injectable PrEP into telehealth delivery models will be important. Given that injectable PrEP cannot be delivered remotely, like oral PrEP can, telehealth systems will need to partner with brick-and-mortar clinics capable of administering injections for PrEP delivery. Thus, successful integration of injectable PrEP into telePrEP care will require operational partnerships and referral pathways to ensure access for patients who select this modality. Emerging models for delivery of injectable PrEP are being explored by researchers and clinicians, including mobile nursing units and visiting nursing services that are equipped to administer injectable PrEP to patients in community and home-care settings [43, 44]. Further implementation research is needed to develop best practices for capitalizing on telehealth’s convenience and accessibility while ensuring availability of new PrEP modalities requiring in-person delivery.

Across the studies, the majority of participants were cisgender men, with the exception of one study focused exclusively on cisgender women. Transgender individuals and gender diverse persons comprised around 2% of the samples in the four studies reporting these demographics. However, no studies specifically focused enrollment on transgender and non-binary communities or provided outcomes specific to these groups. The lack of representation and reporting on transgender and gender diverse participants represents a gap in the literature, especially considering the substantial barriers these communities face regarding access to PrEP services [45, 46]. Understanding and addressing barriers to preventive care services for transgender communities is critical, given the ongoing disparities in HIV burden experienced by these groups driven by social and structural inequities that limit access to prevention and treatment [47–49]. Telehealth holds promise as a way to address some of these barriers to care, especially considering that transgender patients may have experiences and expectations of discrimination in traditional health care settings [50]. Given the existing evidence of the acceptability of telehealth platforms among transgender individuals [51, 52], future research should focus on enrolling transgender and gender diverse participants and measuring acceptability and feasibility outcomes for telehealth delivery of PrEP.

A major gap highlighted by this review is the lack of reporting on the degree to which telePrEP initiatives engage historically marginalized groups who could benefit from PrEP, specifically Black and Latinx communities, transgender populations, and individuals experiencing housing insecurity. Despite efforts to increase access to PrEP, significant disparities in access remain. In 2022, for each new HIV diagnosis among white individuals, there were 36 PrEP users who were white; for Black and Latinx communities, these numbers were 5 and 9 respectively [6]. Additionally, we found limited examples of telePrEP programs that served rural populations, another group which faces ongoing barriers to accessing sexual health services [53]. Efforts to significantly expand telehealth models for PrEP care run the risk of exacerbating disparities if they primarily serve populations who already have substantial access to PrEP services, such as white individuals, those with higher socioeconomic status, and those residing in large urban areas. To assess the equitable impact of telePrEP programs, a shift is needed towards intentionally measuring and optimizing utilization among marginalized populations. Incorporating community partners representing marginalized groups into telePrEP planning, marketing, enrollment, and evaluation could aid more systematic documentation of reach. Partnerships with community-based organizations with trusted ties to local communities could also strengthen the ability of telehealth models for PrEP care to reach those who could benefit from access to PrEP. Tracking and reporting metrics on reach and equity will help telehealth models to advance PrEP equity rather than inadvertently widening gaps.

This review has several limitations that should be considered when interpreting findings on telePrEP models and their outcomes. First, the majority of studies were small pilot interventions. While these small studies have value for initial feasibility testing and model development, larger trials are needed to determine generalizability and scalability of telePrEP approaches. Additionally, while the methodological quality of most included articles was high, many articles were found to have at least one point of methodological weakness through our quality appraisal. Second, studies predominately used observational study designs without control groups for comparison. This significantly limits the ability to draw conclusions about the causal impacts of telehealth delivery on clinical outcomes like PrEP initiation and retention compared to traditional in-person care. Third, restricting our review to published peer-reviewed literature means that unpublished programmatic efforts, as well as programs described in conference abstracts and grey literature, were not captured. There are examples of telePrEP initiatives underway that have not yet produced peer-reviewed findings and were therefore not included in our review, such as the Louisiana TelePrEP program [54]. Fourth, individuals opting into these early telePrEP studies may possess greater personal and technological resources that enable engagement with virtual care. Self-selection likely introduces bias such that reported outcomes reflect experiences of telePrEP early-adopters and may not be representative of the diverse populations that are vulnerable to HIV and who could potentially benefit from PrEP (e.g., individuals experiencing homelessness, recent immigrants, individuals engaging in sex work). In particular, access to private spaces to confidentially discuss medical issues may be a challenge for some groups. Future research should explore how partnering with trusted community-based organizations could help provide the necessary technology, internet access, and private spaces needed to equitably expand access to telePrEP. Finally, the lack of consistent reporting on telePrEP model characteristics and outcome measures across studies limited our ability to compare how specific model components influenced program outcomes. Standardized reporting of intervention components and outcome measures is needed to enable rigorous evaluation of different telePrEP implementation strategies. Future research should prioritize comparative effectiveness designs to directly test the impact of model components on implementation outcomes and clinical outcomes.

This systematic review underscores telehealth’s potential to enhance convenient and patient-centered access to PrEP. Findings demonstrate that telePrEP models tend to show feasibility, acceptability, and moderately successful clinical outcomes that mirror in-person care delivery. However, questions persist regarding optimal implementation strategies, comparative effectiveness to traditional models, and the degree to which telePrEP can promote health equity by extending services to marginalized groups facing barriers to in-person PrEP care. Research and evaluation focused on innovative models of PrEP delivery will be needed to achieve national HIV prevention goals and advance health equity. If developed and tailored to meet the needs of vulnerable communities, telehealth presents a pivotal opportunity to increase PrEP uptake and persistence among those most at-risk for HIV acquisition.

## Electronic Supplementary Material

Below is the link to the electronic supplementary material.

Supplementary Material 1

Supplementary Material 2

## Data Availability

The data that support the findings of this study are available on reasonable request from the corresponding author, SB.

## References

[1] Fauci AS, Redfield RR, Sigounas G, Weahkee MD, Giroir BP. Ending the HIV epidemic: a plan for the United States. JAMA: J Am Med Association. 2019;321:844–5. 10.1001/jama.2019.1343 30730529

[2] Mayer KH, Agwu A, Malebranche D. Barriers to the wider use of pre-exposure Prophylaxis in the United States: a narrative review. Adv Therapy. 2020;37:1778–811. 10.1007/s12325-020-01295-0 PMC7467490 32232664

[3] Laborde ND, Kinley PM, Spinelli M, Vittinghoff E, Whitacre R, Scott HM, et al. Understanding PrEP persistence: provider and patient perspectives. AIDS Behav. 2020;24:2509–19. 32048078 10.1007/s10461-020-02807-3 PMC8054778

[4] Ogunbajo A, Storholm ED, Ober AJ, Bogart LM, Reback CJ, Flynn R, et al. Multilevel barriers to HIV PrEP uptake and adherence among black and Hispanic/Latinx transgender women in southern California. AIDS Behav. 2021;25:2301–15. 33515132 10.1007/s10461-021-03159-2 PMC7845787

[5] Nydegger LA, Dickson-Gomez J, Ko TK. Structural and syndemic barriers to PrEP adoption among black women at high risk for HIV: a qualitative exploration. Cult Health Sex. 2021;23:659–73. 32212993 10.1080/13691058.2020.1720297 PMC7529643

[6] AIDSVu. Deeper Look: PrEP [Internet]. https://aidsvu.org/resources/deeper-look-prep/.

[7] Reisner SL, Moore CS, Asquith A, Pardee DJ, Mayer KH. The pre-exposure prophylaxis cascade in at-risk transgender men who have sex with men in the United States. LGBT Health. 2021;8:116–24. 33567245 10.1089/lgbt.2020.0232 PMC8195872

[8] Siegler AJ, Bratcher A, Weiss KM. Geographic access to preexposure prophylaxis clinics among men who have sex with men in the United States. Am J Public Health. 2019;109:1216–23. 31318587 10.2105/AJPH.2019.305172 PMC6687234

[9] Barnett AP, Arnold T, Elwy AR, Brock JB, Giorlando KK, Sims-Gomillia C, et al. Considerations for PrEP implementation at Federally Qualified Health Centers in Mississippi: perspectives from staff and patients. AIDS Educ Prev. 2023;35:309–19. 37535326 10.1521/aeap.2023.35.4.309 PMC10483574

[10] Calabrese SK, Underhill K. How stigma surrounding the use of HIV preexposure prophylaxis undermines prevention and pleasure: a call to destigmatize truvada whores. Am J Public Health. 2015;105:1960–4. 26270298 10.2105/AJPH.2015.302816 PMC4566537

[11] Giorlando KK, Arnold T, Barnett AP, Leigland A, Whiteley L, Brock JB, et al. Acceptability and comfort regarding remotely delivered PrEP services in Mississippi. J Int Association Providers AIDS Care (JIAPAC). 2023;22:23259582231186868. 10.1177/23259582231186868 PMC10331183 37415442

[12] Shrestha R, Altice FL, Khati A, Azwa I, Gautam K, Gupta S, et al. Clinic-Integrated Smartphone App (JomPrEP) to improve uptake of HIV Testing and Pre-exposure Prophylaxis among men who have sex with men in Malaysia: mixed methods evaluation of usability and acceptability. JMIR mHealth uHealth. 2023;11:e44468. 36795465 10.2196/44468 PMC9982718

[13] Henderson L, Gibbs J, Quinn J, Ramasami S, Estcourt C. Maintaining access to HIV pre-exposure prophylaxis in a pandemic: a service evaluation of telephone-based pre-exposure prophylaxis provision. Int J STD AIDS. 2022;33:718–21. 35465791 10.1177/09564624211068766 PMC9189322

[14] Hoagland B, Torres TS, Bezerra DRB, Benedetti M, Pimenta C, Veloso VG et al. High acceptability of PrEP teleconsultation and HIV self-testing among PrEP users during the COVID-19 pandemic in Brazil. Brazilian Journal of Infectious Diseases [Internet]. 2021;25. https://www.scopus.com/inward/record.uri?eid=2-s2.0-85097770629&doi=10.1016%2fj.bjid.2020.11.002&partnerID=40&md5=3f5a7e05928316b3fc55a66a65ecf09d 10.1016/j.bjid.2020.11.002 PMC7833416 33285137

[15] Siegler AJ, Brock JB, Hurt CB, Ahlschlager L, Dominguez K, Kelley CF, et al. An electronic pre-exposure prophylaxis initiation and maintenance home care system for nonurban young men who have sex with men: protocol for a randomized controlled trial. JMIR Res Protocols. 2019;8:e13982. 10.2196/13982 PMC6592500 31199326

[16] Patel VV, Ginsburg Z, Golub SA, Horvath KJ, Rios N, Mayer KH, et al. Empowering with PrEP (E-PrEP), a peer-led social media–based intervention to facilitate HIV preexposure prophylaxis adoption among young Black and latinx gay and bisexual men: protocol for a cluster randomized controlled trial. JMIR Res Protocols. 2018;7:e11375. 10.2196/11375 PMC6134229 30154071

[17] Patel P, Kerzner M, Reed JB, Sullivan PS, El-Sadr WM. Public Health Implications of Adapting HIV Pre-exposure Prophylaxis Programs for Virtual Service Delivery in the Context of the COVID-19 Pandemic: Systematic Review. JMIR Public Health and Surveillance [Internet]. 2022;8. https://www.scopus.com/inward/record.uri?eid=2-s2.0-85131770671&doi=10.2196%2f37479&partnerID=40&md5=6112dd594267dd7c3dabb517051d8b9e 10.2196/37479 PMC9177169 35486813

[18] Evans KN, Hassan R, Townes A, Buchacz K, Smith DK. The potential of Telecommunication Technology to address Racial/Ethnic disparities in HIV PrEP awareness, Uptake, Adherence, and persistence in care: a review. AIDS Behav. 2022;26:3878–88. 35614366 10.1007/s10461-022-03715-4 PMC9131988

[19] Wong KYK, Stafylis C, Klausner JD. Telemedicine: a solution to disparities in human immunodeficiency virus prevention and pre-exposure prophylaxis uptake, and a framework to scalability and equity. Mhealth. 2020;6. 10.21037/mhealth.2019.12.06 PMC7138949 32270013

[20] Touger R, Wood BR. A review of telehealth innovations for HIV pre-exposure prophylaxis (PrEP). Curr HIV/AIDS Rep. 2019;16:113–9. 30701404 10.1007/s11904-019-00430-z

[21] Moher D, Shamseer L, Clarke M, Ghersi D, Liberati A, Petticrew M, et al. Preferred reporting items for systematic review and meta-analysis protocols (PRISMA-P) 2015 statement. Syst Reviews. 2015;4:1–9. 10.1186/2046-4053-4-1 PMC4320440 25554246

[22] Kohl C, McIntosh EJ, Unger S, Haddaway NR, Kecke S, Schiemann J, et al. Online tools supporting the conduct and reporting of systematic reviews and systematic maps: a case study on CADIMA and review of existing tools. Environ Evid. 2018;7:1–17.

[23] HealthHIV. Telehealth Practitioner’s Guide for HIV Prevention and Care [Internet]. https://www.cdc.gov/hiv/effective-interventions/library/telehealth/implementation-materials/cdc-hiv-ei-telehealth-practitioners-guide.pdf.

[24] Hong QN, Fàbregues S, Bartlett G, Boardman F, Cargo M, Dagenais P, et al. The mixed methods Appraisal Tool (MMAT) version 2018 for information professionals and researchers. Educ Inform. 2018;34:285–91.

[25] Chasco EE, Shafer C, Dillon DMB, Owens S, Ohl ME, Hoth AB. Bringing Iowa TelePrEP to Scale: a qualitative evaluation. Am J Prev Med. 2021;61:S108–17. 34686280 10.1016/j.amepre.2021.05.040

[26] Hoth AB, Shafer C, Dillon DB, Mayer R, Walton G, Ohl ME. Iowa TelePrEP: a public-health-partnered Telehealth Model for Human Immunodeficiency Virus Preexposure Prophylaxis Delivery in a rural state. Sex Transm Dis. 2019;46:507–12. 31295217 10.1097/OLQ.0000000000001017

[27] Hughes SD, Koester KA, Engesaeth E, Hawkins MV, Grant RM. Human Enough: A qualitative study of client experience with internet-based access to pre-exposure prophylaxis. Journal of Medical Internet Research [Internet]. 2021;23. https://www.scopus.com/inward/record.uri?eid=2-s2.0-85109971850&doi=10.2196%2f22650&partnerID=40&md5=f7c2a656a98d0a3fbf87d8eff7716078 10.2196/22650 PMC8406118 36256828

[28] Koester KA, Hughes SD, Grant RM. A Good Habit: Telehealth PrEP Users Find Benefit in Quarterly Monitoring Requirements. Journal of the International Association of Providers of AIDS Care [Internet]. 2020;19. https://www.scopus.com/inward/record.uri?eid=2-s2.0-85083812264&doi=10.1177%2f2325958220919269&partnerID=40&md5=a2b2a36ac768bb81d1e6edca50eb3a39 10.1177/2325958220919269 PMC7180299 32323593

[29] Player MS, Cooper NA, Perkins S, Diaz VA. Evaluation of a telemedicine pilot program for the provision of HIV pre-exposure prophylaxis in the Southeastern United States. AIDS Care. 2022;1–7. 10.1080/09540121.2021.2018567 34978217

[30] Refugio ON, Kimble MM, Silva CL, Lykens JE, Bannister C, Klausner JD. Brief report: PrEPTECH: a telehealth-based initiation program for HIV pre-exposure prophylaxis in young men of color who have sex with men. A pilot study of feasibility. JAIDS J Acquir Immune Defic Syndr. 2019;80:40–5. 30272632 10.1097/QAI.0000000000001873 PMC6291368

[31] Meyer JP, Price CR, Ye Y, Qin Y, Tracey D, Demidont A, et al. A PrEP demonstration project using ehealth and community outreach to justice-involved cisgender women and their risk networks. AIDS Behav. 2022;26:3807–17. 35672552 10.1007/s10461-022-03709-2

[32] Stekler JD, McMahan V, Ballinger L, Viquez L, Swanson F, Stockton J, et al. HIV pre-exposure prophylaxis prescribing through telehealth. JAIDS J Acquir Immune Defic Syndr. 2018;77:e40–2. 29280768 10.1097/QAI.0000000000001621

[33] Bagchi AD, Damas K, Salazar de Noguera N, Melamed B, Menifield C, Baveja A, et al. Comfort with Telehealth among residents of an Underserved Urban Area. J Prim Care Community Health. 2022;13:21501319221119692. 36039812 10.1177/21501319221119692 PMC9434674

[34] Pathela P, Jamison K, Blank S, Daskalakis D, Hedberg T, Borges C. The HIV pre-exposure Prophylaxis (PrEP) cascade at NYC sexual health clinics: navigation is the key to uptake. JAIDS J Acquir Immune Defic Syndr. 2020;83:357–64. 31904700 10.1097/QAI.0000000000002274

[35] Wagner GA, Wu K-S, Anderson C, Burgi A, Little SJ. Predictors of human immunodeficiency Virus Pre-exposure Prophylaxis (PrEP) uptake in a sexual Health Clinic with Rapid PrEP initiation. Open Forum Infectious diseases. Oxford University Press US; 2023. p. ofad060. 10.1093/ofid/ofad060 PMC10034584 36968957

[36] Lankowski AJ, Bien-Gund CH, Patel VV, Felsen UR, Silvera R, Blackstock OJ. PrEP in the real world: predictors of 6-month retention in a diverse urban cohort. AIDS Behav. 2019;23:1797–802. 30341556 10.1007/s10461-018-2296-x PMC6474829

[37] Tao J, Montgomery MC, Williams R, Patil P, Rogers BG, Sosnowy C, et al. Loss to follow-up and re-engagement in HIV pre-exposure prophylaxis care in the United States, 2013–2019. AIDS Patient Care STDs. 2021;35:271–7. 34242092 10.1089/apc.2021.0074 PMC8262386

[38] Kiptinness C, Kuo AP, Reedy AM, Johnson CC, Ngure K, Wagner AD, et al. Examining the Use of HIV Self-Testing to support PrEP delivery: a systematic literature review. Curr HIV/AIDS Rep. 2022;19:394–408. 35904695 10.1007/s11904-022-00617-x PMC9334974

[39] Norelli J, Zlotorzynska M, Sanchez T, Sullivan PS. Scaling up CareKit: lessons learned from expansion of a centralized home HIV and sexually transmitted infection testing program. Sex Transm Dis. 2021;48:S66. 34030160 10.1097/OLQ.0000000000001473 PMC8284343

[40] Russell A, Tasker S, Nichols K, Tweed M, Darking M, Whetham J, et al. Returning home sampling kits for STI and HIV testing in people using a digital health HIV-PrEP pathway (PrEP-EmERGE). Sex Transm Infect. 2023;99:289–90. 36823112 10.1136/sextrans-2022-055724

[41] US Food and Drug Administration. FDA Approves First Injectable Treatment for HIV Pre-Exposure Prevention [Internet]. FDA Newsroom. 2021. https://www.fda.gov/news-events/press-announcements/fda-approves-first-injectable-treatment-hiv-pre-exposure-prevention.

[42] Liegeon G, Ghosn J. Long-acting injectable cabotegravir for PrEP: a game-changer in HIV prevention? HIV Med. 2023;24:653–63. 36468218 10.1111/hiv.13451

[43] White F. Telemedicine bridging urban-rural divide in HIV prevention [Internet]. Oregon, Health. & Science University; 2023. https://news.ohsu.edu/2023/12/01/telemedicine-bridging-urban-rural-divide-in-hiv-prevention.

[44] López González L. South Africa to begin piloting injectable PrEP in early 2023 [Internet]. aidsmap; 2022. https://www.aidsmap.com/news/nov-2022/south-africa-begin-piloting-injectable-prep-early-2023.

[45] Dang M, Scheim AI, Teti M, Quinn KG, Zarwell M, Petroll AE, et al. Barriers and facilitators to HIV pre-exposure prophylaxis uptake, adherence, and persistence among transgender populations in the United States: a systematic review. AIDS Patient Care STDs. 2022;36:236–48. 35687813 10.1089/apc.2021.0236 PMC9242706

[46] Bass SB, Kelly PJ, Brajuha J, Gutierrez-Mock L, Koester K, D’Avanzo P, et al. Exploring barriers and facilitators to PrEP use among transgender women in two urban areas: implications for messaging and communication. BMC Public Health. 2022;22:1–10. 34991548 10.1186/s12889-021-12425-w PMC8740429

[47] Wirtz AL, Humes E, Althoff KN, Poteat TC, Radix A, Mayer KH, et al. HIV incidence and mortality in transgender women in the eastern and southern USA: a multisite cohort study. Lancet HIV. 2023;10:e308–19. 36868260 10.1016/S2352-3018(23)00008-5 PMC10164681

[48] Poteat T, Reisner SL, Radix A. HIV epidemics among transgender women. Curr Opin HIV AIDS. 2014;9:168. 24322537 10.1097/COH.0000000000000030 PMC5947322

[49] Logie CH, James Ll, Tharao W, Loutfy MR. We don’t exist: a qualitative study of marginalization experienced by HIV-positive lesbian, bisexual, queer and transgender women in Toronto, Canada. J Int AIDS Soc. 2012;15:10–7448. 22989529 10.7448/IAS.15.2.17392 PMC3494165

[50] Kcomt L. Profound health-care discrimination experienced by transgender people: Rapid systematic review. Soc Work Health Care. 2019;58:201–19. 30321122 10.1080/00981389.2018.1532941

[51] Apple DE, Lett E, Wood S, Freeman Baber K, Chuo J, Schwartz LA, et al. Acceptability of Telehealth for gender-affirming care in transgender and gender diverse youth and their caregivers. Transgender Health. 2022;7:159–64. 35586576 10.1089/trgh.2020.0166 PMC9051868

[52] Mintz LJ, Gillani B, Moore SE. Telehealth in trans and gender diverse communities: the impact of COVID-19. Curr Obstet Gynecol Rep. 2022;11:75–80. 35463051 10.1007/s13669-022-00334-7 PMC9016376

[53] Valentine JA, Delgado LF, Haderxhanaj LT, Hogben M. Improving sexual health in US rural communities: reducing the impact of stigma. AIDS Behav. 2022;26:90–9. 34436713 10.1007/s10461-021-03416-4 PMC8390058

[54] Louisiana Health Hub. TelePrEP Program [Internet]. https://www.louisianahealthhub.org/teleprep/.

[55] Page MJ, Moher D, Bossuyt PM, Boutron I, Hoffmann TC, Mulrow CD et al. PRISMA 2020 explanation and elaboration: updated guidance and exemplars for reporting systematic reviews. BMJ. 2021;372. 10.1136/bmj.n160 PMC8005925 33781993

[56] NURX. Does Nurx accept health insurance for medication? [Internet]. https://www.nurx.com/faq/does-nurx-accept-health-insurance-for-medication/.

