# Supplementary material for: Telehealth Models for PrEP Delivery: A Systematic Review of Acceptability, Implementation, and Impact on the PrEP Care Continuum in the United States
Source: AIDS Behav. 2024 Jun 10;28(9):2875–86. doi: 10.1007/s10461-024-04366-3 (PMC11390827; doi:10.1007/s10461-024-04366-3)
Supplement: Supplementary file 1 — Supplementary Material 1 [file 10461_2024_4366_MOESM1_ESM.docx]

**Supplemental Figure 1**

| **Database** | **Search Terms** | **Articles Yielded** |
| --- | --- | --- |
| PubMed | (((“PrEP” OR “pre-exposure prophylaxis” OR “preexposure prophylaxis” OR “pre-exposure prophylaxis” OR “chemoprevention” OR “Chemoprophylaxis” OR “iPREX” OR “Truvada” OR “Descovy” OR “tenofovir” OR “emtricitabine” OR “cabotegravir” OR “apretude”) AND (“telemedicine” OR “telehealth” OR “eHealth” OR “e-health” OR “mHealth” OR “m-health” OR “mobile health” OR “mobile technology” OR “mobile applications” OR “app” OR “ap “telemedicine” OR “telehealth” OR “eHealth” OR “e-health” OR “mHealth” OR “m-health” OR “mobile health” OR “mobile technology” OR “mobile applications” OR “app” OR “apps” OR “social medi*” OR “cell phone*” OR “cellphone*” OR “mobile phone*” OR “mobile telephone*” OR “cellular phone*” OR “smartphone*” OR “smart phone*” OR “mobile device*” OR “online” OR “internet” OR “web” OR “digital health” OR “remote*” OR "virtual health" OR "virtual medicine" ps” OR “social medi*” OR “cell phone*” OR “cellphone*” OR “mobile phone*” OR “mobile telephone*” OR “cellular phone*” OR “smartphone*” OR “smart phone*” OR “mobile device*” OR “online” OR “internet” OR “web” OR “digital health” OR “remote*” OR "virtual health" OR "virtual medicine")) OR (“TelePrEP” OR “PrEPTech”)) AND (((((((("knowledge" OR "percept*" OR "understand*" OR "attention" OR "recogni*" OR "familar*" OR "Aware*") OR (“willing*” OR "will")) OR ("intention" OR "intentional" OR "intent" OR "intend")) OR ("Uptake" OR "Usage" OR "Utilization" OR "Increase" OR "interest*" OR “Use”)) OR (“Adherence” OR “electronic monitoring” OR "pill count" OR "TDF/FTC active metabolites" OR "Self-Report" OR "drug concentration" OR "concentration")) OR (“Retention” OR “Compliance” OR “persisten*” OR "followup" OR "follow-up" OR "follow up")) OR (“Accept*” OR "attitude" OR "adoption" OR "appropriate*" OR "Suitability" OR "stigma" OR "stereotype*")) OR (“continu*” OR "feasibility" OR "effective*" OR "practica*")) Date: After January 01 2012 | 1535 |
| APA PsychInfo | (((("PrEP" OR "pre-exposure prophylaxis" OR "preexposure prophylaxis" OR "pre-exposure prophylaxis" OR "chemoprevention" OR "Chemoprophylaxis" OR "iPREX" OR "Truvada" OR "Descovy" OR "tenofovir" OR "emtricitabine" OR "cabotegravir" OR "apretude") AND ("telemedicine" OR "telehealth" OR "eHealth" OR "e-health" OR "mHealth" OR "m-health" OR "mobile health" OR "mobile technology" OR "mobile applications" OR "app" OR "ap " telemedicine " OR " telehealth " OR " eHealth " OR " e-health " OR " mHealth " OR " m-health " OR " mobile health " OR " mobile technology " OR " mobile applications " OR " app " OR " apps " OR " social medi* " OR " cell phone* " OR " cellphone* " OR " mobile phone* " OR " mobile telephone* " OR " cellular phone* " OR " smartphone* " OR " smart phone* " OR " mobile device* " OR " online " OR " internet " OR " web " OR " digital health " OR " remote* " OR " virtual health " OR " virtual medicine " ps" OR "social medi*" OR "cell phone*" OR "cellphone*" OR "mobile phone*" OR "mobile telephone*" OR "cellular phone*" OR "smartphone*" OR "smart phone*" OR "mobile device*" OR "online" OR "internet" OR "web" OR "digital health" OR "remote*" OR "virtual health" OR "virtual medicine")) OR ("TelePrEP" OR "PrEPTech")) AND (((((((("knowledge" OR "percept*" OR "understand*" OR "attention" OR "recogni*" OR "familar*" OR "Aware*") OR ("willing*" OR "will")) OR ("intention" OR "intentional" OR "intent" OR "intend")) OR ("Uptake" OR "Usage" OR "Utilization" OR "Increase" OR "interest*" OR "Use")) OR ("Adherence" OR "electronic monitoring" OR "pill count" OR "TDF/FTC active metabolites" OR "Self-Report" OR "drug concentration" OR "concentration")) OR ("Retention" OR "Compliance" OR "persisten*" OR "followup" OR "follow-up" OR "follow up")) OR ("Accept*" OR "attitude" OR "adoption" OR "appropriate*" OR "Suitability" OR "stigma" OR "stereotype*")) OR ("continu*" OR "feasibility" OR "effective*" OR "practica*"))) AND stype.exact("Scholarly Journals") AND pd(>20120101) | 473 |
| Sociological Abstracts | SUMMARY(((("PrEP" OR "pre-exposure prophylaxis" OR "preexposure prophylaxis" OR "pre-exposure prophylaxis" OR "chemoprevention" OR "Chemoprophylaxis" OR "iPREX" OR "Truvada" OR "Descovy" OR "tenofovir" OR "emtricitabine" OR "cabotegravir" OR "apretude") AND ("telemedicine" OR "telehealth" OR "eHealth" OR "e-health" OR "mHealth" OR "m-health" OR "mobile health" OR "mobile technology" OR "mobile applications" OR "app" OR "ap " telemedicine " OR " telehealth " OR " eHealth " OR " e-health " OR " mHealth " OR " m-health " OR " mobile health " OR " mobile technology " OR " mobile applications " OR " app " OR " apps " OR " social medi* " OR " cell phone* " OR " cellphone* " OR " mobile phone* " OR " mobile telephone* " OR " cellular phone* " OR " smartphone* " OR " smart phone* " OR " mobile device* " OR " online " OR " internet " OR " web " OR " digital health " OR " remote* " OR " virtual health " OR " virtual medicine " ps" OR "social medi*" OR "cell phone*" OR "cellphone*" OR "mobile phone*" OR "mobile telephone*" OR "cellular phone*" OR "smartphone*" OR "smart phone*" OR "mobile device*" OR "online" OR "internet" OR "web" OR "digital health" OR "remote*" OR "virtual health" OR "virtual medicine")) OR ("TelePrEP" OR "PrEPTech")) AND (((((((("knowledge" OR "percept*" OR "understand*" OR "attention" OR "recogni*" OR "familar*" OR "Aware*") OR ("willing*" OR "will")) OR ("intention" OR "intentional" OR "intent" OR "intend")) OR ("Uptake" OR "Usage" OR "Utilization" OR "Increase" OR "interest*" OR "Use")) OR ("Adherence" OR "electronic monitoring" OR "pill count" OR "TDF/FTC active metabolites" OR "Self-Report" OR "drug concentration" OR "concentration")) OR ("Retention" OR "Compliance" OR "persisten*" OR "followup" OR "follow-up" OR "follow up")) OR ("Accept*" OR "attitude" OR "adoption" OR "appropriate*" OR "Suitability" OR "stigma" OR "stereotype*")) OR ("continu*" OR "feasibility" OR "effective*" OR "practica*")))Limits applied Databases: Sociological Abstracts Limited by: Date: After January 01 2012 Narrowed by:Source type: Scholarly Journals | 135 |
| CINAHL | (((“PrEP” OR “pre-exposure prophylaxis” OR “preexposure prophylaxis” OR “pre-exposure prophylaxis” OR “chemoprevention” OR “Chemoprophylaxis” OR “iPREX” OR “Truvada” OR “Descovy” OR “tenofovir” OR “emtricitabine” OR “cabotegravir” OR “apretude”) AND (“telemedicine” OR “telehealth” OR “eHealth” OR “e-health” OR “mHealth” OR “m-health” OR “mobile health” OR “mobile technology” OR “mobile applications” OR “app” OR “ap “telemedicine” OR “telehealth” OR “eHealth” OR “e-health” OR “mHealth” OR “m-health” OR “mobile health” OR “mobile technology” OR “mobile applications” OR “app” OR “apps” OR “social medi*” OR “cell phone*” OR “cellphone*” OR “mobile phone*” OR “mobile telephone*” OR “cellular phone*” OR “smartphone*” OR “smart phone*” OR “mobile device*” OR “online” OR “internet” OR “web” OR “digital health” OR “remote*” OR "virtual health" OR "virtual medicine" ps” OR “social medi*” OR “cell phone*” OR “cellphone*” OR “mobile phone*” OR “mobile telephone*” OR “cellular phone*” OR “smartphone*” OR “smart phone*” OR “mobile device*” OR “online” OR “internet” OR “web” OR “digital health” OR “remote*” OR "virtual health" OR "virtual medicine")) OR (“TelePrEP” OR “PrEPTech”)) AND (((((((("knowledge" OR "percept*" OR "understand*" OR "attention" OR "recogni*" OR "familar*" OR "Aware*") OR (“willing*” OR "will")) OR ("intention" OR "intentional" OR "intent" OR "intend")) OR ("Uptake" OR "Usage" OR "Utilization" OR "Increase" OR "interest*" OR “Use”)) OR (“Adherence” OR “electronic monitoring” OR "pill count" OR "TDF/FTC active metabolites" OR "Self-Report" OR "drug concentration" OR "concentration")) OR (“Retention” OR “Compliance” OR “persisten*” OR "followup" OR "follow-up" OR "follow up")) OR (“Accept*” OR "attitude" OR "adoption" OR "appropriate*" OR "Suitability" OR "stigma" OR "stereotype*")) OR (“continu*” OR "feasibility" OR "effective*" OR "practica*")) Limiters - Published Date: 20120101-20231231 Expanders - Apply equivalent subjects Search modes - Boolean/Phrase Source Type: Academic Journals | 578 |
| Scopus | (((“PrEP” OR “pre-exposure prophylaxis” OR “preexposure prophylaxis” OR “pre-exposure prophylaxis” OR “chemoprevention” OR “Chemoprophylaxis” OR “iPREX” OR “Truvada” OR “Descovy” OR “tenofovir” OR “emtricitabine” OR “cabotegravir” OR “apretude”) AND (“telemedicine” OR “telehealth” OR “eHealth” OR “e-health” OR “mHealth” OR “m-health” OR “mobile health” OR “mobile technology” OR “mobile applications” OR “app” OR “ap “telemedicine” OR “telehealth” OR “eHealth” OR “e-health” OR “mHealth” OR “m-health” OR “mobile health” OR “mobile technology” OR “mobile applications” OR “app” OR “apps” OR “social medi*” OR “cell phone*” OR “cellphone*” OR “mobile phone*” OR “mobile telephone*” OR “cellular phone*” OR “smartphone*” OR “smart phone*” OR “mobile device*” OR “online” OR “internet” OR “web” OR “digital health” OR “remote*” OR "virtual health" OR "virtual medicine" ps” OR “social medi*” OR “cell phone*” OR “cellphone*” OR “mobile phone*” OR “mobile telephone*” OR “cellular phone*” OR “smartphone*” OR “smart phone*” OR “mobile device*” OR “online” OR “internet” OR “web” OR “digital health” OR “remote*” OR "virtual health" OR "virtual medicine")) OR (“TelePrEP” OR “PrEPTech”)) AND (((((((("knowledge" OR "percept*" OR "understand*" OR "attention" OR "recogni*" OR "familar*" OR "Aware*") OR (“willing*” OR "will")) OR ("intention" OR "intentional" OR "intent" OR "intend")) OR ("Uptake" OR "Usage" OR "Utilization" OR "Increase" OR "interest*" OR “Use”)) OR (“Adherence” OR “electronic monitoring” OR "pill count" OR "TDF/FTC active metabolites" OR "Self-Report" OR "drug concentration" OR "concentration")) OR (“Retention” OR “Compliance” OR “persisten*” OR "followup" OR "follow-up" OR "follow up")) OR (“Accept*” OR "attitude" OR "adoption" OR "appropriate*" OR "Suitability" OR "stigma" OR "stereotype*")) OR (“continu*” OR "feasibility" OR "effective*" OR "practica*")) Date: After January 01 2012 | 95 |
| Total |  | 2816 |
