# Supplementary material for: Telehealth Models for PrEP Delivery: A Systematic Review of Acceptability, Implementation, and Impact on the PrEP Care Continuum in the United States
Source: AIDS Behav. 2024 Jun 10;28(9):2875–86. doi: 10.1007/s10461-024-04366-3 (PMC11390827; doi:10.1007/s10461-024-04366-3)
Supplement: Supplementary file 2 — Supplementary Material 2 [file 10461_2024_4366_MOESM2_ESM.docx]

Supplemental Table 1: Quality appraisal of included studies using the Mixed Methods Appraisal tool (MMAT)

|  |  | **Screening questions** | | **Qualitative** | | | | | **Quantitative non-randomized** | | | | | **Quantitative descriptive** | | | | | **Mixed methods** | | | | |
| --- | --- | --- | --- | --- | --- | --- | --- | --- | --- | --- | --- | --- | --- | --- | --- | --- | --- | --- | --- | --- | --- | --- | --- |
|  | **Study Design** | Q1 | Q2 | Q3 | Q4 | Q5 | Q6 | Q7 | Q13 | Q14 | Q15 | Q16 | Q17 | Q18 | Q19 | Q20 | Q21 | Q22 | Q23 | Q24 | Q25 | Q26 | Q27 |
| **Chasco et al. (2021) [24]** | Mixed Methods (Qualitative + Quantitative descriptive) | Y | Y | Y | Y | Y | Y | Y |  |  |  |  |  | Y | Y | Y | Y | Y | Y | ? | Y | Y | Y |
| **Hoth et al. (2019) [25]** | Quantitative Descriptive | Y | Y |  |  |  |  |  |  |  |  |  |  | Y | Y | Y | Y | Y |  |  |  |  |  |
| **Hughes et al. (2021) [26]** | Qualitative | Y | Y | Y | Y | Y | N | Y |  |  |  |  |  |  |  |  |  |  |  |  |  |  |  |
| **Koester et al. (2020) [27]** | Qualitative | Y | Y | Y | Y | ? | ? | N |  |  |  |  |  |  |  |  |  |  |  |  |  |  |  |
| **Player et al. (2022) [28]** | Quantitative Descriptive | Y | Y |  |  |  |  |  |  |  |  |  |  | Y | N | Y | Y | Y |  |  |  |  |  |
| **Refugio, et al. (2019) [29]** | Quantitative Descriptive | Y | Y |  |  |  |  |  |  |  |  |  |  | Y | Y | Y | Y | Y |  |  |  |  |  |
| **Meyer et al. (2022) [30]** | Quantitative Descriptive | Y | Y |  |  |  |  |  |  |  |  |  |  | Y | Y | N | Y | Y |  |  |  |  |  |
| **Stekler et al.  (2018)  [31]** | Quantitative non-randomized | Y | Y |  |  |  |  |  | Y | Y | Y | N | Y |  |  |  |  |  |  |  |  |  |  |
